# Indications for total-body computed tomography in blunt trauma patients: a systematic review

**DOI:** 10.1007/s00068-016-0711-4

**Published:** 2016-07-19

**Authors:** K. Treskes, T. P. Saltzherr, J. S. K. Luitse, L. F. M. Beenen, J. C. Goslings

**Affiliations:** 1 Trauma Unit, Department of Surgery, Academic Medical Center, Meibergdreef 9, 1105 AZ Amsterdam, The Netherlands; 2 Department of Radiology, Academic Medical Center, Amsterdam, Meibergdreef 9, 1105 AZ Amsterdam, The Netherlands

**Keywords:** Total-body CT, Whole body imaging, Multiple trauma, Wounds and injuries, Computed tomography

## Abstract

**Purpose:**

Total-body CT scanning (TBCT) could improve the initial in-hospital evaluation of severe trauma patients. Indications for TBCT, however, differ between trauma centers, so more insight in how to select patients that could benefit from TBCT is required. The aim of this review was to give an overview of currently used indications for total-body CT in trauma patients and to describe mortality and Injury Severity Scores of patient groups selected for TBCT.

**Methods:**

A systematic review was performed by searching MEDLINE and Embase databases. Studies evaluating or describing criteria for selection of patients with potentially severe injuries for TBCT during initial trauma care were included. Also, studies comparing total-body CT during the initial assessment of injured patients with conventional imaging and selective CT in specific patient groups were included.

**Results:**

Thirty eligible studies were identified. Three studies evaluated indications for TBCT in trauma with divergent methods. Combinations of compromised vital parameters, severe trauma mechanisms and clinical suspicion on severe injuries are often used indications; however, clinical judgement is used as well. Studies describing TBCT indications selected patients in different ways and were difficult to compare regarding mortality and injury severity.

**Conclusions:**

Indications for TBCT in trauma show a wide variety in structure and cut-off values for vital parameters and trauma mechanism dimensions. Consensus on indications for TBCT in trauma is lacking.

**Electronic supplementary material:**

The online version of this article (doi:10.1007/s00068-016-0711-4) contains supplementary material, which is available to authorized users.

## Introduction

The work-up of trauma patients by ATLS (advanced trauma life support) guidelines uses a step-up approach for diagnostic imaging. After conventional radiography of the chest and pelvis and focused assessment by sonography (FAST), selective computed tomography can be performed subsequently on indication [1]. Ongoing improvements in speed and accuracy of computed tomography (CT) and increased availability of CT scanners in or nearby the trauma room made immediate total-body CT (TBCT) feasible as a diagnostic tool in the initial assessment of trauma patients. Initial trauma care, thus, might be improved when total-body CT scan is incorporated in the initial assessment of a potentially multiple and severely injured patient [2].

A disadvantage of TBCT scanning is increased radiation exposure for patients that appear to have minor injuries for which selective CT scanning on indication could be sufficient. For the overall group of trauma patients, the proportion of patients receiving a high radiation dose of >20 mSv at the trauma room is increased [3]. For multitrauma patients, the radiation dose is, however, comparable for the complete hospital admission [4]. To prevent excessive radiation exposure, the appropriate selection of patients for TBCT is essential [3, 5]. The decision to perform an immediate TBCT is based on information obtained during the pre-hospital phase and the first in-hospital assessment. Therefore, indications such as compromised vital parameters, clinical suspicion on severe injuries and high-risk injury mechanisms are often used to select trauma patients that might benefit from immediate TBCT.

Justification for performing a TBCT is only possible in hindsight, when all diagnoses have been confirmed by radiologic imaging, interventions and the clinical course. Moreover, different outcome measures are used to justify TBCT, such as: classification as multiple or severely injured patient by anatomical scoring systems (e.g., Injury Severity Score) or certain high-risk profiles for injuries [6–8]. To improve selection and to guide future research on the proper indications for TBCT after major trauma, a better insight in current indications is required. Therefore, the aim of this review was (1) to give an overview of currently used indications for total-body CT in trauma patients and (2) to describe mortality and Injury Severity Scores of patient groups selected for TBCT.

## Methods

For this systematic review, the preferred reporting items for systematic reviews and meta-analyses (PRISMA) are used as a guideline [9].

### Inclusion and exclusion criteria

Studies evaluating or describing indications for TBCT during initial trauma care were included. Also, studies comparing TBCT during the initial assessment of injured patients with conventional imaging and selective CT in specific patient groups were included. TBCT should at least comprise the following body regions: head, neck, thorax, abdomen and pelvis. For selection of studies, no distinction was made between immediate TBCT and TBCT with preceding conventional radiologic imaging. Reviews, randomized and observational studies describing original data were eligible for inclusion. Study protocols, case reports and editorials were excluded. Literature in a language other than English or German was also excluded.

### Search strategy

The MEDLINE and Embase library databases were searched for articles published between 1947 and July 2014. The search terms consisted of synonyms of ‘total-body CT’ combined with synonyms and words related to trauma and injury. The full search is presented in Supplementary Appendix 2. The last search was performed in July 2014 and was conducted with the help of a clinical librarian. A cross-reference search was performed on the included articles.

### Study selection and data extraction

Two reviewers independently assessed titles and abstracts of all studies identified by the initial search and excluded irrelevant studies. Second, the full texts of the remaining eligible studies were assessed to determine whether they met the inclusion criteria. Any discrepancies in inclusion were resolved by discussion between the reviewers. In case no consensus was reached, this was solved by a third reviewer. The following data from each included paper were extracted: author, publication year, country, study design, inclusion criteria, sample size, Injury Severity Score (ISS), indications for TBCT, and outcome.

## Results

### Study selection

**Fig. 1:**
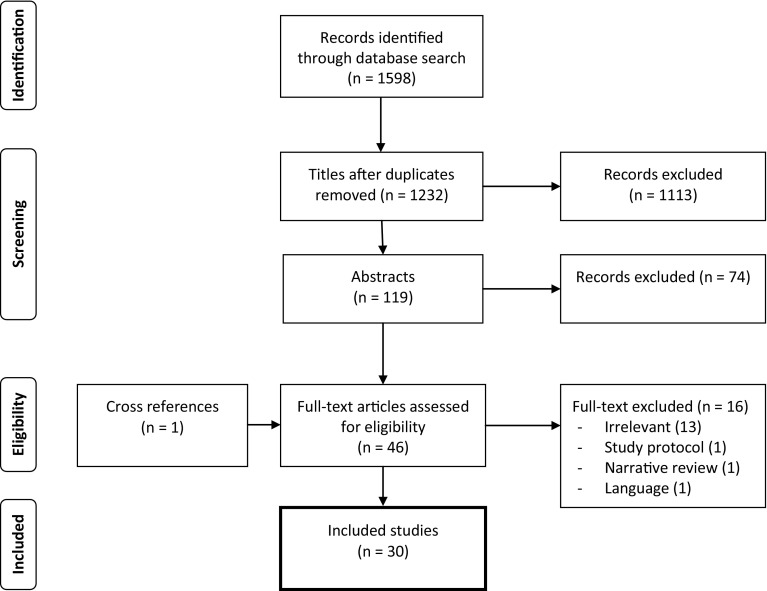
Flowchart for the selection of studies

The search identified 532 records from the MEDLINE database and 1006 records from the Embase database. 366 duplicates were removed. 30 studies were included for data extraction (Fig. 1). Included study designs were retrospective for 17 studies and prospective or observational for 10 studies. The remaining three were a randomized clinical trial, a case-matched study and one questionnaire survey. Studies were published between 2003 and 2013, except for one, which was published in 1998.

### Studies on TBCT indications

For three included studies, the main objective was to evaluate indications for TBCT in trauma patients. Wurmb et al. [8] assessed whether a triage scheme could appropriately select sedated and ventilated patients with severe trauma for TBCT scanning. This triage scheme used specific trauma mechanisms, compromised vital signs and clinically obvious injuries. An Injury Severity Score (ISS) of 16 or higher was used to define severe trauma. Sensitivity of this triage scheme for severe trauma was 96.7 % and positive predictive value was 69.4 %.

Hsiao et al. [7] also used an anatomical definition of severe trauma to justify TBCT for patients that triggered trauma team activation and were CT-scanned during the initial in-hospital assessment. An Abbreviated Injury Score (AIS) of 2 or more in two or more body regions defined multi-regional injury. Clinical judgement had a sensitivity of 50 % and a 32 % positive predictive value for multi-region injury. Mean ISS was 17 (SD16) for patients that underwent TBCT. Multivariable logistic regression resulted in the following independent predictors for multi-region injury: full trauma team activation, GCS <9, fall >5 m or pedal cyclist. The derived prediction model did not show an improvement for accuracy of selection when compared to decision by clinical judgement.

Babaud et al. [6] evaluated the French national triage criteria [10] (Vittel criteria) for detecting patients with at least one injury. Multivariable logistic regression within the patient group resulted in the following independent predictors for detection of an injury: GCS <13, penetrating trauma and resuscitation with >1000 mL colloids. For 15 % of the patients selected by one or more Vittel criteria, an unsuspected severe injury was detected by TBCT.

### Characteristics of populations studied to assess the effect of TBCT

Seventeen studies reported their indications for TBCT in trauma. Sets of indications consisted of combinations of compromised vital parameters (15 studies), high-risk trauma mechanisms (14 studies), clinical suspicion of severe injury (12 studies) and clinical judgement (2 studies). In eight other studies, the decision to perform a TBCT was based only on clinical judgement or suspicion on severe or multiple injuries. Table 1 further shows the patient population, ISS, type of indications used for TBCT and the outcome measures for the included studies.

**Table 1:** Overview of included studies

Author, study year, country	Study design	Patients overall (TBCT)	ISS, median (IQR) TBCT/control/overall	TBCT indications	Outcome
Sierink 2014, The Netherlands [14]	CM	304 (152)	18 (9–29)/18 (8–29)/NA	VP, CSI	30d Mortality
Wada 2013, Japan [21]	RS	152 (132)	34 (25–45)/41 (34–51)/NA	CJ	28d SMR (TRISS)
Sierink 2013, The Netherlands [4]	RS	301 (151)	22 (18–27)/25 (17–29)/NA	VP, CSI	Radiation exposure
Huber-Wagner 2013, Germany [11]	RS	16,719 (9233)	30 (12)/28 (12)/29 (12)^a^	Not defined	SMR (RISC)
Sedlic 2013, Canada [13]	RS	67 (67)	NA	VP, TM, CSI	SMR (TRISS)
Kimura 2013, Japan [22]	RS	5208 (1858)	26 (25–26)/23 (23–24)/NA^a^	VP: GCS	SMR (TRISS)
Hsiao 2013, Australia [7]	RS	660 (98)	17 (16)/5 (6)/NA^a^	CJ/PM: VP, TM, FTTA	Multi-region injured^c^
Asha 2012, Australia [3]	RS	1280 (624)	4 (2–10)/4 (2–10)/4 (2–10)	VP, TM/CJ, CSI	Radiation exposure/missed injuries
Babaud 2012, France [6]	PS	339 (189)	NA	VP, TM, CSI (Vittel)	Unsuspected injuries
Stengel 2012, Germany [23]	RS	982 (982)	25 (18–33)/-/25 (18–33)	VP, TM, CSI, CJ (DGU)	Missed injuries
Hutter 2011, Germany [24]	OS	1144 (608)	21 (9)/28 (12)/NA^a^	VP, TM	Mortality
Gupta 2011, USA [17]	PS	701 (600)	5 (1–14)/2 (1–5)/5 (1–13)	CJ	Missed injuries
Smith 2011, UK [25]	OS	254 (138)	14 (11)/7 (6)/NA^a^ 13 (11)/7 (9)/NA	TM	Change of treatment
Wurmb 2011, Germany [26]	RS	318 (163)	27 (17–41)/24 (13–34)/NA	VP, TM, CSI (Nast-Kolb)	Time to surgery/mortality
Smith 2012, UK [27]	Survey	245 hospitals	–	VP, TM, CSI, PMI, WS	–
Tillou 2009, USA [18]	PS	284	13 (1–17)/-/13 (1–17)	CJ	Unsuspected injuries
Huber-Wagner 2009, Germany [2]	RS	4621 (1494)	32 (14)/28 (12)/30 (13)^a^	Not defined	SMR (TRISS/RISC)
Wurmb 2009, Germany [28]	RS	161 (82)	24 (11–33)/22 (11–32)/NA	VP, TM, CSI (Nast-Kolb)	Time to diagnosis
Rieger, 2009, Austria [16]	RS	88	29 (10)/-/29 (10)^a^	VP, TM, CSI (Nast-Kolb)	Time to diagnosis/missed injuries
Nguyen 2009, Swiss [29]	OS	90	NA	TM	Examination time
Wurmb 2007, Germany [8]	RS	120 (85)	NA/NA/19 (3–75)	VP, TM, CSI (Nast-Kolb)	Polytrauma (ISS ≥16)
Weninger 2007, Austria [15]	OS	370 (185)	27 (10)/28 (12)/NA^a^	Not defined	Accuracy/time to diagnosis
Prokop 2006, Germany [12]	RS	100	33 (12)/-/33 (12)^a^	CJ	Examination time
Salim 2006, USA [19]	PS	1000	NA	Normal abdominal PE, and TM	Change of treatment
Sampson 2006, UK [30]	RS	296	NA	Not defined	(unsuspected) injuries
Wurmb 2005, Germany [31]	PC	120 (78)	NA	VP, TM, CSI (Nast-Kolb)	Examination time
Heyer 2005, Germany [32]	RCT	80	NA	CJ	Examination time/radiation exposure
Albrecht 2004, Germany [33]	RS	50	NA	CJ	Missed injuries
Self 2003, USA [20]	RC	457	NA	CJ	Change of treatment
Leidner 1998, Sweden [34]	PS	111	NA	CJ	Examination time/missed injuries

*ISS* Injury Severity Score, *IQR* interquartile ranges, *CM* case-matched study, *RS* retrospective study, *PM* prediction model, *OS* observational study, *PS* prospective study, *RCT* randomized clinical trial, *VP* vital parameters, *TM* trauma mechanism, *CSI* clinical suspicious injury, *CJ* clinical judgement, *FTTA* full trauma team activation, *PE* physical examination, *SMR* standardized mortality ratio

^a^Mean, SD

^b^Mean, 95 % CI

^c^Multi-region injured defined by AIS ≥2 in ≥2 body regions (head/face, vertebral column, chest, abdomen/pelvis)

Table 2 shows that selection of multitrauma patients was often a result of the study design rather than selection of patients for TBCT by trauma leaders. Five retrospective studies enrolled patients with an ISS of 16 or higher [2, 11–14]. Weninger et al. [15] included only patients with an ISS of 17 or higher and at least one body region with an AIS of 4 or higher. Rieger et al. [16] included patients with an ISS of 18 or higher. Two prospective studies included patients who triggered trauma team activation and reported a median ISS of 5 (IQR 1–14) and 13 [1–17] for patients who underwent TBCT based on clinical judgement [17, 18]. Hsiao et al. [7] retrospectively selected patients receiving CT imaging during trauma assessment and reported a mean ISS of 17 (SD16) for patients with an indication for TBCT by clinical judgement. The remaining studies that described an indication by clinical judgement, retrospectively selected patients by ISS or bleeding control measures (Table 2).

**Table 2:** Overview of reported mortality and polytrauma proportion in populations selected for TBCT studies

Author, study year, country	Eligibility criteria besides blunt trauma, adult and direct transfer	Mortality (%) TBCT/control/overall	Polytrauma, ISS ≥16 (%) TBCT/control/overall
Sierink 2014, The Netherlands [14]	≥1 VP or CSI	13.0/13.0/13.0 (30d)	63.2/63.2/63.2
Wada 2013, Japan [21]	Requiring bleeding control	18.1/80.0/26.3 (28d)	>75/>75/>75
Sierink 2013, The Netherlands [4]	ISS ≥16 and ≥1 VP or CSI	5.3/4.6/5.0 (30d)	100 (by protocol)
Huber-Wagner 2013, Germany [11]	ISS ≥16	17.4/21.4/19.2 (overall)	100 (by protocol)
Sedlic 2013, Canada [13]	TBCT performed, and ISS ≥16, and ≥1 VP, TM or CSI	14.9/-/- (ND)	100 (by protocol)
Kimura 2013, Japan [22]	GCS 3–12, SBP >75 mmHg	24/28/27 (ND)	NA
Hsiao 2013, Australia [7]	Trauma team activation and initial CT scan required	3.1/1.2/1.5 (ND)	51.5/16.5/21.7
Asha 2012, Australia [3]	Trauma team activation	NA	17.5/18.5/18.0
Babaud 2012, France [6]	≥1 Vittel criterion	NA	NA
Stengel 2012, Germany [23]	≥ 1 VP, TM or CSI, CJ	7.1/-/7.1 (ND)	36.7
Hutter 2011, Germany [24]	Admission to trauma center	15/8/13 (overall)	95.1/96.9/95.5
Gupta 2011, USA [17]	Trauma team activation after blunt trauma	NA	–/–/20
Smith 2011, UK [25]	Suspicion on having multiple or serious injuries	4.7 (ND)	NA
Wurmb 2011, Germany [26]	(suspected) Multiple trauma requiring emergency surgery	5.8/5.5/5.7 (30d)	87.1/71.6/84.4
Smith 2012, UK [27]	–	–	–
Tillou 2009, USA [18]	Trauma team activation after blunt trauma	NA	NA
Huber-Wagner 2009, Germany [2]	ISS ≥16	21/22/22 (overall)	100 (by protocol)
Wurmb 2009, Germany [28]	ISS ≥18	NA	100 (by protocol)
Rieger, 2009, Austria [16]	Treatment in resuscitation area by trauma team	NA	67.0/58.2/62.7
Nguyen 2009, Swiss [29]	TBCT performed, and MVC or fall from >3 m	NA	NA
Wurmb 2007, Germany [8]	Sedated and ventilated trauma patients	NA	69.4/5.7/50.8
Weninger 2007, Austria [15]	ISS ≥17, and AIS ≥4 in ≥1 body region (head, thorax or abdomen), and survival until ICU admission	17/16/17	100 (by protocol)
Prokop 2006, Germany [12]	ISS >16 and TBCT performed	13/-/13	100 (by protocol)
Salim 2006, USA [19]	No visible evidence of chest or abdominal injury, and hemodynamically stable, and PE of abdomen normal or unevaluable because of depressed level of consciousness, and significant mechanism of injury	NA	NA
Sampson 2006, UK [30]	Hemodynamically stable, and AIS ≥2 in ≥1 body region (head/neck, thorax, abdomen/pelvis, spine or extremities)	NA	NA
Wurmb 2005, Germany [31]	Treatment in resuscitation area by trauma team	NA	NA
Heyer 2005, Germany [32]	Suspected injury of ≥2 body regions of which ≥1 is life-threatening, and ICU admission	NA	NA
Albrecht 2004, Germany [33]	Prehospital suspected polytrauma, and TBCT performed	NA	NA
Self 2003, USA [20]	Blunt head injury and TBCT performed	NA	NA
Leidner 1998, Sweden [34]	Hemodynamically stable, and clinical suspicion of multiple organ injuries or a trauma mechanism capable of producing major injury to multiple organ systems.	NA	NA

*ISS* Injury Severity Score, *VP* vital parameters, *TM* trauma mechanism, *CSI* clinical suspicious injury, *CJ* clinical judgement, *ND* not defined, *NA* not available

In the appendix, the described TBCT indications after trauma and cut-off values for vital parameters and trauma mechanism dimensions are presented from 30 included articles. These are categorized by vital parameters, clinical suspicious injuries, high-risk trauma mechanism and contraindications. For all the included literature, minor age and isolated penetrating injury were formulated as contraindications for TBCT or indirectly formulated by including only adult patients sustaining blunt trauma.

## Discussion

In this systematic review of studies that evaluate or describe indications for TBCT in initial trauma care, we showed similarities and differences of these indications. There is a wide variety of eligibility criteria and outcome measures between studies (Table 2). Combinations of compromised vital parameters, severe trauma mechanisms and clinical suspicion on severe injuries are most often reported, however, clinical judgement on expected severe and multiple injuries is described as well. Within these groups of indications, there is a large variation in used parameters and cut-off values (Supplementary Appendix 1). Because of this variety between sets of indications, it is difficult to compare indications for TBCT between studies.

Differences in outcome measures for justification of TBCT in hindsight implicate a lack of consensus toward patient groups that rightfully received a TBCT during their trauma work-up. Anatomical scoring systems with different thresholds for ISS and AIS for body regions are used to justify the performance of TBCT or to select patients who might benefit from TBCT scanning [7, 8]. Several retrospective studies on TBCT select patients by anatomical scoring systems and, therefore, suggest that patients above these thresholds could benefit from TBCT. Other outcome measures reflecting the severity or extent of injuries might be suitable as well, such as mortality, morbidity, ICU admittance, surgical and radiological interventions or detection of unsuspected injuries.

Not only parameters reflecting severe injury could justify TBCT. Decreased levels of consciousness could be considered an indication on itself since clinical indicators for imaging are unreliable owing to the lack of subjective input from the patient. Routine CT imaging for patients with unreliable physical examination is reported to reveal unsuspected findings in up to 38 %, leading to treatment changes in 19–26 % [19, 20]. Furthermore, one could hypothesize that TBCT might lead to early discharge for less severely injured patients when used to rule out injuries [19]. Since the probability of detecting injuries after major trauma during the clinical course of alert patients might be lowered after TBCT, the in-hospital observation of the clinical course might be less valuable.

This review included only three studies for which the main objective was to evaluate indications for TBCT in trauma patients. Studies that described mortality and ISS already chose study eligibility criteria to select patients that might benefit from TBCT. Thereby, the wide variety of eligibility criteria made comparison of mortality and ISS of patient groups selected for TBCT less valuable. Besides limited comparability of methods, there was also a low availability of mortality and ISS for the included studies.

An anatomical scoring system such as ISS as indication for TBCT cannot be used in daily practice, because the results are calculated after radiologic imaging is performed. As well as other outcome parameters reflecting severe injury, anatomical scoring systems could only be helpful as an outcome measure for the evaluation of the indication for TBCT and not to define the indication for TBCT.

In this overview of TBCT indications, we did not make a distinction between immediate TBCT and TBCT after conventional X-rays and sonography. Future prospective research on the indication for one or both strategies should consider this difference in its design. Furthermore, there was no distinction made regarding different imaging protocols. Contrast enhancement and body position were not described for included studies.

Little is known of the predictive value of specific parameters within the sets of indications for severe and multiple injury. However, reduced Glasgow Coma Scale (GCS) after major trauma seems to be a valid indication for TBCT. First, it is reported to independently predict multi-region injury and detection of injury, in general. Second, the unreliability of the physical examination can result in unsuspected findings needing treatment. Decision for a cut-off value for GCS might depend on which goal one pursues: to select multiple and severely injured patients or reduction of missed injuries after major trauma.

Future research needs to prospectively determine the positive predictive value of separate TBCT indications for multiple and severely injured patients. Positive predictive values for TBCT indications are useful for determining the proportion of patients that were appropriately selected for TBCT, and the concomitant radiation exposure could, therefore, be accepted. To determine the proportion of the multiple and severely injured patients selected for TBCT, sensitivity of a set of indications has to be calculated. Emphasis on specific diagnostic tests changes when another type of outcome measure is chosen such as reduction of missed injuries.

The question remains as to whether we should use fixed sets of indications for TBCT, and, if so, how they should be defined. In the meantime, one should be aware that selection of patients for TBCT by clinical judgement alone could result in relatively low ISS. Independently from which outcome measure is chosen, one should carefully weigh the potential benefits of TBCT to an increased radiation exposure and potential increase of costs. The unsuspected findings and eventual shortening of hospital admission should outweigh the increased radiation exposure to make TBCT beneficial for the less severely injured patients.

## Conclusion

Indications for TBCT in trauma show a wide variety in formulation and cut-off values for vital parameters and trauma mechanism dimensions. Combinations of compromised vital parameters, severe trauma mechanisms and clinical suspicion on severe injuries are often used. However, clinical judgement on expected severe and multiple injury is used as well. Consensus on outcome measures for justification of TBCT should be obtained to guide further research on the appropriate indications for TBCT in trauma.

## Electronic supplementary material

Below is the link to the electronic supplementary material.

Supplementary material 1 (DOC 71 kb)

Supplementary material 2 (DOC 55 kb)
