# Supplementary material for: Indications for total-body computed tomography in blunt trauma patients: a systematic review
Source: Eur J Trauma Emerg Surg. 2016 Jul 19;43(1):35–42. doi: 10.1007/s00068-016-0711-4 (PMC5306321; doi:10.1007/s00068-016-0711-4)
Supplement: Supplementary file 1 — Supplementary material 1 (DOC 71 kb) [file 68_2016_711_MOESM1_ESM.doc]

**Appendix 1**

Overview of described TBCT indications after trauma and cut-off values for vital parameters and trauma mechanism dimensions

| Type of indication | Subtype | Cut-off value |
| --- | --- | --- |
| Vital Parameters | - Respiratory rate - O_2_ Saturation - Pulse - Systolic blood pressure - Estimated exterior blood loss - GCS - Abnormal pupillary reaction on site | ≥/> 29/min or </≤ 10/min  <90% / <85% for age >75yr  ≥ 120/min / <50/min  <80 / <90 / ≤100 / <100 for age > 65yr  ≥ 500 ml  <9 / <10 / ≤ 13 / ≤ 14 for age >65yr |
| Trauma mechanism | - Fall - Motor vehicle (driver, passenger)   - Initial speed >65 kph (40 mph) / 35mph   - Combined velocitiy ≥ 50km/h   - High speed crash   - Major auto deformity >50 cm (20 in.)   - Intrusion into passenger compartment >30 cm (12 in.)   - Vehicle rollover   - head-on collision   - Ejection from vehicle / car   - Extrication time >20 min   - Entrapment > 30 minutes / trapped in car   - Crush injury to thorax/abdomen   - Death same passenger compartment - Pedestrian struck   - By motor vehicle at any speed   - With significant impact >10 kph (5 mph)   - Thrown >10 ft or run over - Bicyclist struck   - Hit by larger vehicles   - Hit by car   - With significant impact >10 kph (5 mph) - Motorcyclist   - High speed crash   - Crash >30 kph (20 mph) / >50kph   - Separation from motorcycle - Victim thrown or run over - Crash against truck - Technical rescue required / extrication - Global assessment (vehicle deformation, estimated speed, no helmet, no seat belt) - Major industrial accident - Blast injury / explosion, buried person - Significant assault - Assaulted with depressed level of consciousness - Torso crush injury - Unknown mechanism with abnormal vital parameters - Unknown mechanism | >3m / > 5m / >6m / unclear height |
| Clinically suspicious injury | - Fractures of ≥ 2 (proximal) long bones - Flail chest, open chest, or multiple rib fractures - (unstable) pelvic fracture - Smashed pelvis - Open abdominal wound - Unstable vertebral fractures - Spinal cord injury / suspected spinal cord injury - Penetrating injuries to head, neck, chest, abdomen, groin,and extremities proximal to elbow and knee - Penetrating injury - Gunshot wound (including air rifle) - Stabwound - Combination trauma with burns >20 % of BSA - Severe burn, smoke inhalation - Amputation proximal to wrist and ankle - Crush injury proximal to wrist and ankle - Traumatic limb paralysis - Acute ischemia of a limb - Any evidence of airway obstruction or compromise - Multiple body region injuries |  |
| Clinical judgement | - Suspected injury of ≥ 2 body regions of which ≥ 1 is life threatening - Suspicion of severe trauma by paramedics or emergency doctors on scene |  |
| Other | - (modified) Early warning score - Requiring bleeding control measurement - Resuscitation prior to admission (assisted ventilation, colloid fluids >1L, catecholamines, inflated antishock trousers) - Predisposition , to be determined (Age>65 years, heart or coronary failure, respiratory failure, 2^nd^ or 3^rd^ trimester pregnancy, dyscrasia) |  |
| Exclusion | - Minor age - pregnancy - Referred from another hospital - too unstable to undergo a CT scan and requires (cardiopulmonary) resuscitation or immediate operation - availability of CT scanner - clear identification of abnormalities by FAST and X-ray - focal / isolated trauma without potential multiple trauma or severe kinetic component as defined by Vittel criteria - Obesity >200kg | < 15, < 17, <18 years |
