# Supplementary material for: Indications for total-body computed tomography in blunt trauma patients: a systematic review
Source: Eur J Trauma Emerg Surg. 2016 Jul 19;43(1):35–42. doi: 10.1007/s00068-016-0711-4 (PMC5306321; doi:10.1007/s00068-016-0711-4)
Supplement: Supplementary file 2 — Supplementary material 2 (DOC 55 kb) [file 68_2016_711_MOESM2_ESM.doc]

**Appendix 2**. Complete search

Medline, 20140717 (532 hits)

(computed tomography protocol*[tw] OR ct protocol[tw] OR msct protocol[tw] OR "Whole Body Imaging"[Mesh] OR whole body imaging[tw] OR whole body scan*[tw] OR whole body screening*[tw] OR total body ct[tw] OR total body scan*[tw] OR (total body[tw] AND computed tomography[tw]) OR total body screening*[tw] OR full body ct[tw] OR (full body[tw] AND computed tomography[tw]) OR full body scan*[tw] OR full body screening*[tw] OR whole body ct[tw] OR whole body computed tomography[tw] OR whole body scan*[tw] OR whole body screening*[tw] OR pan computed[tw] OR fbct[tw] OR fb ct[tw] OR tbct*[tw] OR tb ct[tw] OR additional body[tw]) AND ("Traumatology"[Mesh] OR trauma*[tw] OR polytrauma*[tw] OR "Wounds and Injuries"[Mesh] OR injur*[tw] OR "Shock"[Mesh] OR shock[tw] OR shockroom[tw] OR "Intensive Care Units"[Mesh] OR icu[tw] OR intensive care unit*[tw] OR emergenc*[tw] OR "Emergency Service, Hospital"[Mesh])

Embase 1947 to present, OvidSP, 20140717 (1066 hits)

1. (computed tomography protocol? or ct protocol? or msct protocol? or whole body imaging or whole body scan* or whole body screening* or (total body adj2 ct) or total body scan* or (total body adj2 computed) or total body screening* or (full body adj2 ct) or (full body adj2 computed) or full body scan* or full body screening* or (whole body adj2 ct) or (whole body adj2 computed) or whole body scan* or whole body screening* or pan computed or tbct* or tb ct or additional body).ab,kw,ti.

2. whole body imaging/ or whole body ct/ or whole body tomography/

3. 1 or 2

4. exp injury/ or traumatology/ or shock/ or intensive care unit/ or emergency/ or emergency care/ or emergency health service/

5. (trauma* or polytrauma* or injur* or shock or shockroom or icu or intensive care unit? or emergenc*).ab,kw,ti.

6. 4 or 5

7. 3 and 6
